# Acupuncture for benign prostatic hyperplasia: A systematic review and meta-analysis

**DOI:** 10.1371/journal.pone.0174586

**Published:** 2017-04-04

**Authors:** Wei Zhang, Liyan Ma, Brent A. Bauer, Zhishun Liu, Yao Lu

**Affiliations:** 1 Department of Acupuncture, Guang’anmen Hospital, China Academy of Chinese Medical Sciences, Beijing, China; 2 Clinical Laboratory Center. Beijing Friendship Hospital, Capital Medical University, Beijing, China; 3 Department of Medicine. Mayo Clinic, Rochester, Minnesota, United States of America; Northwestern University, UNITED STATES

## Abstract

**Purpose:**

This systematic review and meta-analysis aims to assess the therapeutic and adverse effects of acupuncture for benign prostatic hyperplasia (BPH) in randomized controlled trials (RCTs).

**Methods:**

We searched the Cochrane Central Register of Controlled Trials (CENTRAL) in The Cochrane Library, MEDLINE, EMBASE, the Chinese Biomedical Database, the China National Knowledge Infrastructure, the VIP Database and the Wanfang Database. Parallel-group RCTs of acupuncture for men with symptomatic BPH were included. Data from the included trials were extracted by two independent reviewers and were analyzed with The Cochrane Collaboration Review Manager software (RevMan 5.3.5) after risk of bias judgments. The primary outcome measure of this review was a change in urological symptoms.

**Results:**

Eight RCTs, which involved 661 men with BPH, were included. Follow-up varied from 4 weeks to 18 months. Pooling of the data from three trials that compared acupuncture with sham-acupuncture revealed that in the short term (4–6 weeks), acupuncture can significantly improve IPSS (MD -1.90, 95% CI -3.58 to -0.21). A sensitivity analysis of the short-term endpoint showed the same result (MD -3.01, 95% CI -5.19 to -0.84) with a borderline minimal clinical important difference (MCID). Qmax of the short-term endpoint indicated statistically positive beneficial effects of acupuncture (MD -1.78, 95%CI -3.43, -0.14). A meta-analysis after medium-term follow-up (12–18 weeks) indicated no significant effect on IPSS when the data from two trials were combined (MD -2.04, 95% CI -4.19, 0.10).

**Conclusion:**

Statistically significant changes were observed in favor of acupuncture in moderate to severe BPH with respect to short-term follow-up endpoints. The clinical significance of these changes needs to be tested by further studies with rigorous designs and longer follow-up times.

**Trial registration number:**

PROSPERO CRD42014013645.

## Introduction

Benign prostatic hyperplasia (BPH) is the most commonly encountered urologic disease among older men. Studies have reported the prevalence of BPH to be approximately one-quarter of men in their 50s, one-third in their 60s, and about half in their 80s [1, 2]. Interventions for BPH include minimally invasive therapies, surgical therapies and medical therapies (including those derived from plants, which is known as phytotherapy) [3, 4]. Acupuncture is a therapy characterized by the stimulation of certain anatomical points (acupoints) using diverse techniques, such as penetration of the skin, with different types of needles. With a literary history of more than 2000 years [5], acupuncture has always been a routine practice in China. In addition, traditional Chinese medicine (TCM) books have revealed the way in which acupuncture can be used to treat diseases [6]. Currently, to some extent, acupuncture has become a popular practice worldwide. Both a WHO report and a National Institutes of Health consensus conference have provided lists of diseases that could be potentially managed with acupuncture [7, 8].

In China, an increasing number of clinical utilizations of acupuncture have been discovered for BPH [9]. A systematic review published in 2010 compared acupuncture versus Western medicine for BPH [10]. However, the insufficient quantity and unsatisfactory quality of the reviewed studies restricted its value to that of a clinical reference. Thus far, comprehensive evidence collections and analyses concerning the effects and safety of acupuncture for BPH are still scant.

The primary aim of this systematic review was to evaluate the effect of acupuncture on changes in urological symptoms and to assess any adverse effects.

## Methods

The protocol used in this systematic review and meta-analysis was published previously with the registration number PROSPERO CRD42014013645 (see S1 Protocol) [11]. A Preferred Reporting Items for Systematic Reviews and Meta-Analyses (PRISMA) checklist is available as supporting information; see S1 Checklist.

### Search strategy

We searched the following electronic databases: the Cochrane Central Register of Controlled Trials (CENTRAL) in The Cochrane Library, MEDLINE, EMBASE, the Chinese Biomedical Database, the China National Knowledge Infrastructure, the VIP Database and the Wanfang Database. We also searched the references of all included studies, bibliographic references in urological textbooks and those in previous reviews. Table 1 shows the full electronic search strategy for MEDLINE. This search strategy can also be seen in S1 Protocol [11]. The date of the search was 1 Sept 2016.

**Table 1:** Search strategy for Ovid MEDLINE.

	Search items
1	randomized controlled trial
2	controlled clinical trial
3	randomised
4	randomized
5	randomly
6	trial
7	or/1-6
8	benign prostatic hyperplasia or bph/
9	lower urinary tract symptoms or luts/
10	or/8-9
11	acupuncture/
12	acupuncture points/
13	(electroacupuncture or electro- acupuncture).tw.
14	electroacupuncture.tw.
15	acupuncture$.tw.
16	acupoints.tw.
17	meridians/
18	or /11-17
19	7 and 10 and 18

### Inclusion and exclusion criteria

Parallel-group RCTs of acupuncture for men with symptomatic BPH were included. Non-RCTs and uncontrolled clinical trials such as case studies were excluded. Acupuncture with needle insertions into the body was included as an intervention for the experimental group. Control intervention included no intervention, placebo/sham acupuncture [12, 13], surgery, minimally invasive therapies or pharmacological treatments. A minimum follow-up of 4 weeks (from trial baseline assessment) was required for inclusion in the analysis.

We did not include trials that used Chinese patent medicine (tablet, pill or capsule) as comparators due to insufficient evidence of their clinically meaningful effect on LUTS secondary to BPH. Most Chinese patent medicines are compounds, and their clinical usages are primarily based on ancient Chinese medical theories. Although these medicines have broad empirical applications and a considerable number are included in publications in China, we chose not to include them as active controls until explicit pharmacologic explanations for their critically active ingredients can be provided.

### Outcome measures

Primary outcome measures of this systematic review were changes in urological symptoms as measured by validated urological symptom scores. Primary and secondary outcome measures, as well as adverse events to be recorded, have been described in detail in our protocol (see S1 Protocol).

### Study selection

Two independent reviewers (LM, YL) explored whether the studies meet the inclusion criteria by screening their titles and abstracts and reading the full texts. Quasi-RCTs and studies that involved unqualified interventions were excluded. A third party (ZL) resolved disagreements via a discussion with responsible reviewers. Study selection was summarized in a PRISMA flow diagram [14].

### Data extraction and managements

Data of variables described in the protocol were assessed and extracted independently by two reviewers (LM, YL). A data extraction form was used to collect data before they were entered into the software of The Cochrane Collaboration Review Manager (RevMan 5.3.5) for calculation of the meta-analysis. We attempted to obtain necessary information by contacting the corresponding authors of the included trials by email when data were missing.

Data of variables included the age of the patients, interventions of the treatment and control groups (type, frequency, dosage and treatment course), as well as primary and secondary outcomes. The type, number and severity of adverse events were also recorded.

### Risk of bias assessment

A tool introduced in the Cochrane Handbook for Systematic Reviews of Interventions (V.5.1), which includes five sources of bias in clinical trials and their relevant domains, was used to assess the risk of bias. Specific features of the included studies were judged independently by two reviewers (YL, LM) in each entry of a “risk of bias” table [15]. Review authors neither assessed the risk of bias nor conducted data extraction for any studies on which they were an author.

### Data synthesis

Data were combined and analyzed with RevMan 5.2.3. We tested for statistical heterogeneity using a standard χ^2^ test with a significance level of p<0.1, and an I^2^ test was used for quantifying inconsistency among the included studies [16]. A fixed-effects model was used for evidence of homogeneity (p>0.1) while a random-effects model was used if there was substantial (I^2^>75%) statistical heterogeneity. Continuous data was expressed as the mean differences (MD) with 95% confidence intervals (CIs). A narrative synthesis was provided if the meta-analysis could not be performed for some of the expected data from the included studies.

### Unit of analysis

Neither cluster-randomized trials nor cross-over trials were included. In case unit of analysis issues arose in studies of long duration, time frames were defined as 4–6, 12–18 and ≥24 weeks to reflect short-term, medium-term and long-term follow-up, respectively.

### Sensitivity analysis

We implemented sensitivity analyses to explore the impacts of evidence quality on the robustness of review conclusions. The meta-analysis was repeated after the removal of studies with a high risk of bias.

### Subgroup analysis

We planned to compare the effects between subgroups according to the following method: 1 Different acupuncture types: Long needle (≥75 mm in length) and regular needle (<75 mm in length) as defined by ancient Chinese medicine literature [17]. 2 Different control interventions, such as Western medicine and phytotherapy.

## Results

### Search results

**Fig 1:**
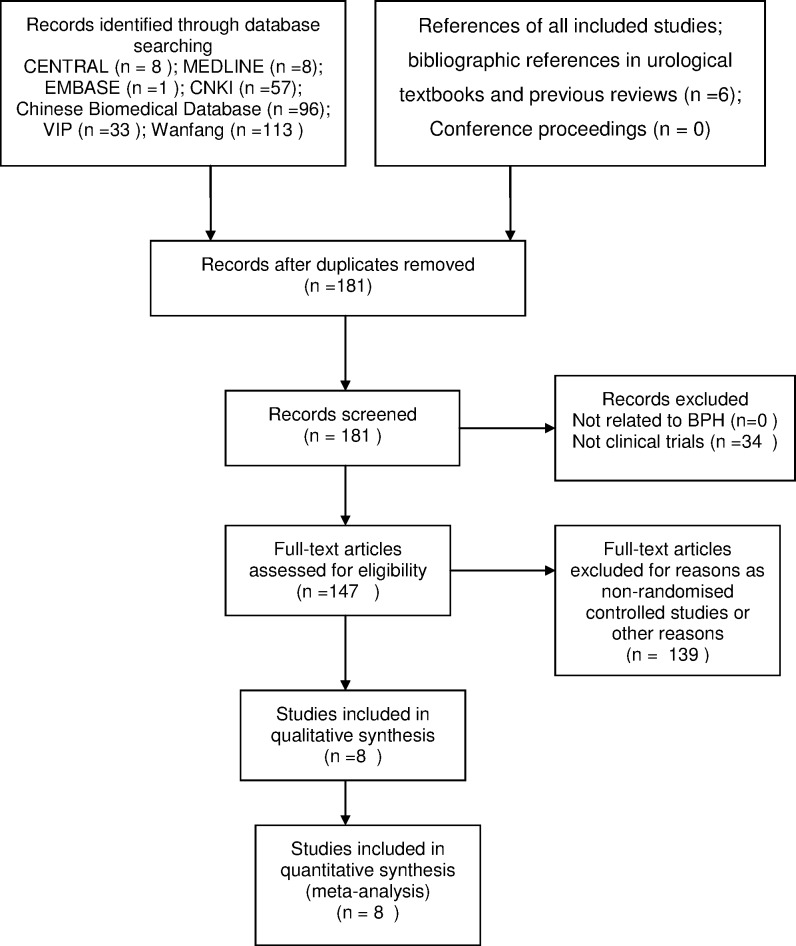
PRISMA flow diagram.

Fig 1 shows the results of the literature searches and the screening process used in this review. Most manuscripts were written in Chinese (305/322). After the duplicates were removed, we identified 176 unique studies from database searches and 5 manuscripts through manual checking of the references of the included studies and related systematic reviews [10]. After the articles were reviewed by title and abstract, we evaluated the full text of 147 records. Finally, 139 studies were excluded from the review. The supporting file describes the reasons of exclusion; see S2 File: List of full-text excluded articles.

### Included studies

We included 8 RCTs, 6 of which were published in Chinese and 2 were published in English [18–25]; these studies involved 661 men with BPH (sample size 42–175). All studies were randomized at the patient level. Follow-up varied from 4 weeks to 18 months. During the procedure of inclusion, nine emails were sent to request further information on published manuscripts (all in Chinese) and only one response was received [22].

All RCTs involved men with diagnosed symptomatic BPH. Five trials defined severity by IPSS as >8 or ≥8 [18,19,20,22,25]. Acupuncture interventions were performed using electro-acupuncture with the exception of one trial that used hand acupuncture [25]. Five trials [18, 20, 22, 23, 24] selected acupoints in the sacrococcygeal area (BL 33 and BL 35) while others selected acupoints of the belly or limbs [19, 21, 25]. Long needles that were greater than 75 mm in length were used in three trials [18, 20, 22], all of which selected the same sacrococcygeal acupoints (bilateral BL33) with electro-acupuncture. Regular needles (40–65 mm) were used in the remaining 5 trials. Sham acupuncture comparators (two non-acupoints [18, 20] and one non-acupoint with shallow insertion [19]) were included in three trials. Five trials compared acupuncture with Western medicine, which consisted of alpha-blockers [21–23] and 5- alpha-reductase inhibitors (5-ARIs) [24, 25]. Acupuncture interventions in all trials were declared to be implemented by qualified acupuncturists. Characteristics of the included studies are described in S1 Table. A description of the studies is also included in the review.

### Risk of bias judgments

**Fig 2:**
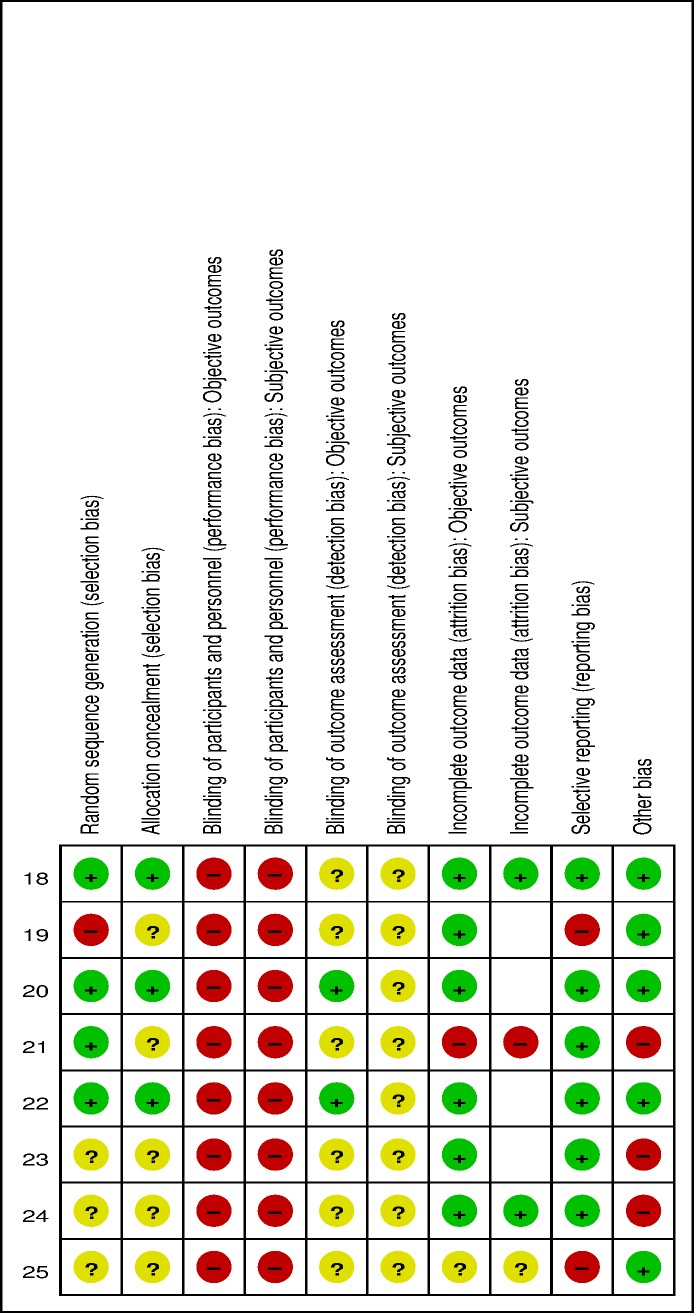
Risk of bias for included trials.

Fig 2 shows the risk of bias judgments for the included studies. The supporting file describes these judgments in detail; see S1 File: Risk of bias judgments. All trials were judged to have a high risk of bias with respect to the blinding of participants given that it is not possible to blind the acupuncturists and most patients in a study of acupuncture intervention compared with medication. However, two trials that involved acupuncture at BL33 and non-acupoints sham acupuncture comparators did blind patients, as they received acupuncture while prone, which limited the ability of the participants to differentiate between sham vs verum treatment [18, 20]. The most common issues that lead to a high risk of bias that could impact study quality were a lack of blinding of the outcome assessment and adverse events reports.

### Results of individual studies

All eight included trials measured the International Prostate Symptom Score (IPSS) [18–25] while two trials measured the Bother Score (BS) [22, 23]. By comparison, these trials were classified into one of three groups: 1 acupuncture versus sham acupuncture [18–20]; 2 acupuncture versus alpha-blockers [21–23]; 3 acupuncture versus 5-ARIs [24–25].

Three trials compared acupuncture versus sham acupuncture [18–20]. After an assessment of the heterogeneity, data from these studies were combined for the meta-analysis (see meta-analysis section).

Three trials compared acupuncture versus alpha-blockers [21– 23] with regard to short-term endpoints. No combination could be made because two trials included patients with mild BPH with an IPSS<8 either in the control group [21] or in both groups [23]; these patients should be managed using a strategy of watchful waiting (active surveillance) according to the American Urological Association Guideline: Management of BPH (revised 2010) [26]. This introduced clinical heterogeneity, which prevented the combination of these studies. One study with a proper definition of severity found better improvement of the IPSS and BS after 4 weeks after, 6 months and 18 months after acupuncture compared with the administration of terazosin hydrochloride tablets [22].

One study compared acupuncture versus 5-ARIs and measured the IPSS with respect to short-term endpoints [24]. One study compared acupuncture with 5-ARIs and measured the IPSS in terms of medium-term follow-up endpoints (3 months) [25]. Positive beneficial effects of acupuncture were found in both trials.

Other secondary outcomes included prostate volume (PV) [21–25], postvoid residual urine (PVR) [18, 20–25], prostate-specific antigen (PSA), total flow time and total void volume [19] and quality of life (QOL) [18, 21, 24, 25]. None of these outcomes were combined.

Six studies reported information on adverse events. Two studies reported no adverse events for the acupuncture group [20, 22], and only one study reported one case of depression and fatigue after acupuncture intervention, which was later diagnosed as male menopausal syndrome [18]. One study reported that, after acupuncture, two patients experienced an urge to defecate, which was resolved after three days without any treatment [23]. One study reported two cases of mild hematoma after sham acupuncture intervention, which was resolved in about two weeks with ice and heat compression [20].

### Meta-analysis

Three trials involved 190 men who were then included in a meta-analysis to assess acupuncture versus sham acupuncture [18–20].

#### Short-term follow-up (IPSS)

**Fig 3:**
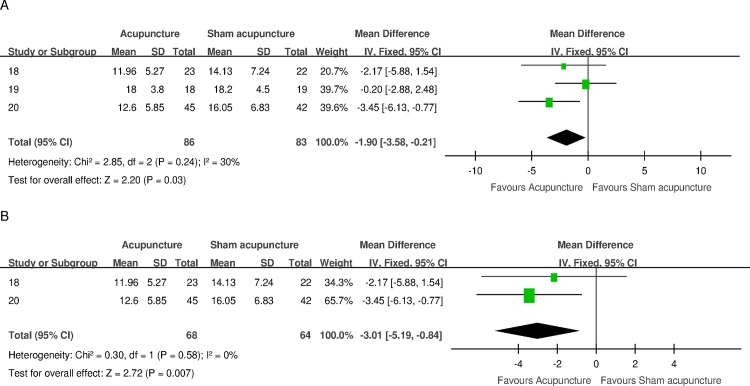
Forest plot of the IPSS for acupuncture versus sham acupuncture for short-term follow-up endpoints. (A) Forest plot of the IPSS for the 3 included trials. (B) Forest plot of the sensitivity analysis of IPSS for the 2 trials of higher quality. SD = standard deviation; CI = confidence interval; df = degrees of freedom.

Three trials that compared acupuncture versus sham acupuncture reported IPSS with respect to short-term follow-up endpoints (4–6 weeks). The difference was statistically significant in favor of acupuncture (MD -1.90, 95% CI -3.58 to -0.21) but was considered below the minimum clinically important difference (MCID) of three points according to the American Urological Association Guideline: Management of BPH (revised 2010) [26]. No statistical heterogeneity was observed (I^2^ 30%). A sensitivity analysis of studies that were judged to be of higher quality [18, 20] indicated a statistically positive effect estimate and a borderline MCID (MD -3.01, 95% CI -5.19 to -0.84) with very low heterogeneity (I^2^ 0%). Fig 3 shows a forest plot of the results.

#### Medium-term and long-term follow-up (IPSS)

**Fig 4:**
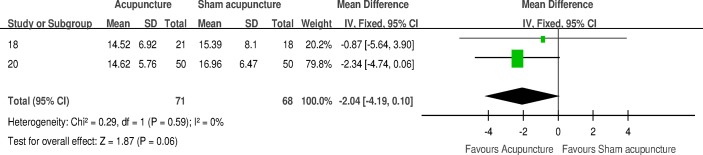
Forest plot of the IPSS for acupuncture versus sham acupuncture for medium-term follow-up endpoints.

Two trials comparing acupuncture versus sham acupuncture reported IPSS on medium-term follow-up points (12–18 weeks) [18, 20]. No statistically significant effect on this outcome was found from combining the data from these two trials (MD -2.04, 95% CI –4.19, 0.10). Very low heterogeneity was observed (I^2^ 0%). Fig 4 shows a forest plot of the results.

#### Maximum flow rate (Qmax)

**Fig 5:**
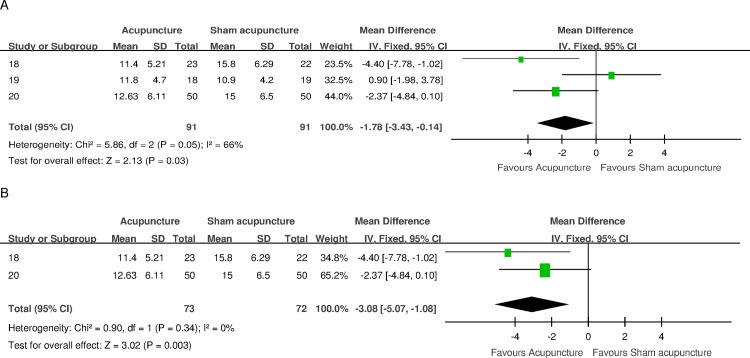
Forest plot of Qmax for acupuncture versus sham acupuncture for short-term follow-up endpoints. (A) Forest plot of Qmax for the 3 trials included. (B) Forest plot of the sensitivity analysis of Qmax for the 2 trials of higher quality.

Three trials measured Qmax on short-term follow-up points (4–6 weeks) concerning acupuncture versus sham acupuncture comparison [18–20]. A statistically positive effect on this outcome was found from combining these studies (MD -1.78, 95%CI -3.43, - 0.14) without substantial heterogeneity (I^2^ 66%). The 2010 National Institute for Health and Care Excellence LUTS in Men Guideline examined the threshold of what constituted the MCID for flow rate changes as 2 mL/s [27]. Sensitivity analysis of studies that were judged to have higher quality [18, 20] indicated a statistically positive effect estimate and a difference above MCID (MD -3.08, 95% CI -5.07,-1.08) with very low heterogeneity (I^2^ 0%). Fig 5 shows a forest plot of the results.

### Publication bias

Analysis of funnel plots is a practical test to detect potential publication bias in meta-analyses. However, the quantity of included trials (8 trials) was too small to conduct any test of publication bias.

### Planned subgroup analysis

We did not perform a subgroup analysis due to an insufficient quantity (three) of included trials for which we conducted a meta-analysis and that determined IPSS outcome. Only one out of the three included studies used a regular needle with acupoints in a location other than the sacrococcygeal area [19].

## Discussion

Eight trials involving 661 men with BPH were included in this review. As a traditional Chinese therapy, acupuncture is dependent on the experience of the acupuncturist so that the correct location of the acupoints and the correct manipulations are guaranteed. All included trials claimed adequate qualification of acupuncture providers. We did not penalize trials for their high risk of performance bias with respect to the blinding of participants, as it is not possible to blind all acupuncturists and patients who participate in trials with a control group other than sham acupuncture by certain methods. However, some trials did successfully blind patients by selecting acupoints located on the body outside of the participants’ field of vision.

Sham control methods for acupuncture clinical trials include the use of placebo needles, the use of non-acupoints (points out of meridians) and the application of shallow needle insertions. The placebo needle introduced by Streitberger in 1998 is a retractable needle with a blunt tip that is held on points by a specially designed needle holder and plastic stand [12]. This needle provides only a pricking sensation when used and does not penetrate the skin. No trial included in this review used this placebo needle, which may have been because of the high expense. Despite that all of these controls have critics and are not universally accepted as “good” controls (e.g., shallow needling might still have positive effects, needling off points might still stimulate substance P, etc.), at this stage, the placebo/sham control is still the first option that can be used to demonstrate the efficacy of acupuncture. Trials that used acupuncture/electro-acupuncture alone as the experimental intervention were included because other TCM therapies such as cupping, moxibustion, plum blossom needling and Chinese herbs, which were mentioned as “co-interventions” in standards for the reporting of interventions in controlled trials of acupuncture (STRICTA) [28], are actually parallel therapies in the TCM system with an effectiveness equivalent to that of acupuncture. Poor reporting of allocation concealment is common. No included trial used a password- protected computer database. The majority of included trials ignored the necessity of independent investigators. Adverse events reports were absent in some studies, which is a significant flaw given that the reporting of adverse events should be standard in any interventional clinical trial. No severe adverse event was reported (e.g., death, pneumothorax). Other adverse events such as fatigue and the sensation of the urge to defecate after acupuncture were either unrelated to the intervention or were mild and quickly reversible.

Data from three trials were combined into a meta-analysis to compare acupuncture with sham-acupuncture. In the short term (4–6 weeks), acupuncture intervention can statistically and significantly improve the IPSS in men with BPH. A sensitivity analysis revealed a difference in borderline MCID (Fig 3). No significant effect on this outcome was found from pooling the data on medium-term (12–18 weeks) endpoints from two trials (Fig 4). According to a previously published report, sham acupuncture (non-acupoints) had better efficacy than conventional treatment for some conditions [29]. This result implies that sham acupuncture may induce active biological effects other than merely placebo effects caused by patient expectancy and hope for recovery. Thus, the MCID between acupuncture and sham acupuncture may be smaller than that between acupuncture and no intervention or placebo medication. Nonetheless, it may be just a speculation that acupuncture can clinically improve LUTS secondary to BPH. No definite conclusion can be drawn due to the scant number of trials.

As a secondary outcome measure, Qmax on short-term endpoints indicated a statistically significant difference that favors acupuncture (Fig 5). However, only one trial [19] reported a baseline Qmax below 10 mL/s for the acupuncture group, which is more suggestive of an obstructed state. Though Qmax is an efficient measure that estimates the probability of an urodynamic obstruction, a low Qmax does not distinguish between obstruction and decreased detrusor contractility. Therefore, this result may suggest that acupuncture can either release obstruction or increase detrusor contractility. Although flow rate recording was frequently utilized among the included trials, few contained a clear diagnosis of benign prostatic obstruction (BPO) with further tests. A pressure flow study is the gold standard for the diagnosis of bladder outlet obstruction [30]. To explore the mechanism of action of acupuncture in BPH, future studies should consider the inclusion of a pressure flow study, at least in a subset of subjects.

Alpha-blockers and 5-ARIs are proper active controls for acupuncture studies. However, subjects should be selected from suitable populations with caution. Inappropriate inclusion of patients with mild symptoms into trials leads to the impossibility of combining data for a meta-analysis. Acupuncture is a complex medical system that typically individualizes treatment based on symptoms and individual characteristics of the patient. This is in contradistinction to Western medicine approaches, which typically focus on the disease and provide a standard treatment based on the diagnosis. Western medicine approaches are also relatively unmindful to the characteristics of the individual patient. Therefore, to effectively design future clinical trials of acupuncture for BPH, active collaborations between acupuncturists and urologists are critical. Since there is insufficient evidence to support the clinical importance of verum acupuncture for BPH, active control with conventional medication should not be strongly recommended, and a non-inferiority design should be adopted.

A previous systematic review of acupuncture for BPH was published in Chinese, and it considered the efficacious rate from inconsistent criteria as the only outcome measure, which makes this study impractical and ambiguous [10]. Our review selected only trials that reported continuous data, mostly for objective outcomes, to support reproducibility. This review and meta-analysis offers the most up-to-date and unique evidence in English on improvements in urological symptoms as well as adverse effects, which were systematically screened from a comprehensive review of RCTs.

Acupoint specificity means that certain point(s) are more effective when used than non-acupoint(s) or other acupoint(s). It has been demonstrated that sacral acupuncture was effective in the improvement of urologic symptoms in animal models [31]. Due to the point selection principles of TCM, local points are generally believed to have reliable effectiveness for conditions manifested in the same area of the human body. It would have been possible for us to implement a subgroup analysis to compare whether a difference exists between acupoints in the sacrococcygeal area and other locations if an adequate number of studies were available. Considering the anatomical characteristics of the sacrococcygeal area, most acupoints located there are good for long needles (so-called “mang” needles in Chinese) with deeper insertion. Animal experiments revealed that to treat urinary retention after spinal cord injury, acupuncture with long needles at BL54 was better than regular acupuncture [32]. Needle type, including related safety implications, is another factor that future studies should consider.

### Limitations

Our analysis has several limitations. First, in spite of the fact that two trials were published in English [19, 20], the populations involved in the included trials were all Chinese. No multi-centered trial with patients of different races was gathered and thus acupuncture treatment for non-Asian populations is still speculative. Second, most included trials (7/8) had follow-up periods no longer than 3 months. Considering that BPH is a progressive disease with long-lasting events, a study with a follow-up less than 24–48 months may not appropriately evaluate the possible effect of a treatment strategy. Third, more outcome measures need to be introduced and analyzed as patients experience acute urinary retention or undergo surgery related to BPH. To discover the possible mechanism of acupuncture for BPH, necessary diagnostic tests in line with existing diagnostic guidelines need to be applied [33, 34]. Fourth, the meta-analysis was confined to acupuncture versus sham acupuncture with a limited number of trials included. Comparative studies that involve active medications as comparators with non-inferiority designs are needed.

## Conclusion

Statistically significant changes were observed in short-term follow-up endpoints in favor of acupuncture in moderate to severe BPH. However, it is not certain whether clinically significant outcomes can be achieved by acupuncture. We recommend future studies to assess sham/placebo acupuncture with reasonable methods, such as control intervention, to determine acupoints or needle type specificity for BPH. Whenever possible, a multi-centered study involving patients of more races and regions should be performed. An acupuncture study with active comparators of medication should be implemented synchronously using good inclusion criteria and strict informed consent.

## Supporting information

S1 Checklist PRISMA Checklist. (PDF)

S1 Protocol Trial Protocol. (PDF)

S1 File Risk of bias judgments. (DOC)

S2 File List of full-text excluded articles. (DOC)

S1 Table Description of studies included in the review. (DOC)
