# Supplementary material for: Acupuncture for benign prostatic hyperplasia: A systematic review and meta-analysis
Source: PLoS One. 2017 Apr 4;12(4):e0174586. doi: 10.1371/journal.pone.0174586 (PMC5380320; doi:10.1371/journal.pone.0174586)
Supplement: S1 File — (DOC) [file pone.0174586.s003.doc]

**RISK OF BIAS JUDGEMENTS**

**Allocation**

**Random sequence generation**

Four studies reported enough information for a judgement of low risk regarding randomisation sequence generation (Liu 2012; Wang 2013; Zheng 2016; Yang 2010). One trial reported randomisation by lottery but can’t provide further detail as whether the lottery was put back into the pool after drawing, which made it judged to be of high risk [Yu 2011]. In all other trials, insufficient information was present to permit judgement.

**Allocation concealment**

Three studies reported enough information for a judgement of low risk allocation concealment (Liu 2012; Wang 2013; Yang 2010). In all other trials, insufficient information was present to permit judgement.

**Blinding**

**Blinding of participants and personnel**

All studies were judged to have a high risk of performance bias, as it would not be possible to blind acupuncturists in an acupuncture intervention and most patients in an acupuncture intervention compared with medication. However, trials with acupuncture at BL33 with sham acupuncture comparators did blind patients for they received acupuncture with face down and little difference can be told from patients standpoints ( Liu 2012, Wang 2013)

**Blinding of outcome assessment**

IPSS and Qmax were reported in all eight included trials. Other trials reported variable numbers of objective outcomes including PV, PSA, BS, PVR, total flow time and total void volume. Four trials reported only objective outcomes (Yu 2011, Wang 2013, Yang 2010, Yu 2005). Being the only subjective outcome, quality of life was reported in four trials (Liu 2012, Zheng 2016, Wang 2006, Du 2012).

Only one trial reported sufficient blinding for assessment of objective outcomes [Wang 2013]. Another trial responded to our enquiry as using blinded statistician to assess objective outcomes [Yang 2010]. Six other studies collecting objective outcomes (Liu 2012, Yu 2011, Zheng 2016, Yu 2005, Wang 2006, Du 2012) reported insufficient information to permit a judgment on bias.

No trial that used subjective outcome (QOL) reported any form of blinding for assessment.

**Incomplete outcome data**

Only one trial failed to report sufficient information for attrition bias judgement [Du 2012]. One trial was judged to have a high risk of attrition bias reporting objective and subjective outcomes (20%) [Zheng 2016]. Other six trials, either reported objective outcomes only [Jung 2011, Wang 2013, Yang 2010, Yu 2005] or objective with subjective outcomes [Liu 2012, Wang 2006] were judged to have low risk of attrition bias (<20%).

**Selective reporting**

All eight trials included reported all specified outcomes of interest in the review. Six studies reported adverse events (Liu 2012; Wang 2013; Zheng 2016; Yang 2010; Yu 2005; Wang 2006). We judged the two trials which failed to report adverse events (Yu 2011; Du 20124) as having a high risk of bias.

**Other potential sources of bias**

Three trials were judged to have high risk of other biases (Zheng 2016; Yu 2005; Wang 2006) for they failed to define BPH severity by IPSS when including subjects. These three trials took western medication as comparators and all of them include men with IPSS<8 either in control group (Zheng 2016) or in both groups (Yu 2005; Wang 2006). According to American Urological Association Guideline: Management of BPH (revised 2010), patients with mild symptoms of LUTS secondary to BPH (AUA-SI score <8) and patients with moderate or severe symptoms (AUA-SI score ≥8) who are not bothered by their LUTS should be managed using a strategy of watchful waiting (active surveillance). Absence of severity definition also introduced heterogeneity which prevent these trials from being entered into a meta-analysis.

References

Liu X. Study on electroacupuncture efficacy and specificities of BL33 (Zhong Liao) for treatment of benign prostatic hyperplasia. M.Sc. Thesis, China Academy of Chinese Medical Sciences. 2012. Available from: http:// www.cnki.net/KCMS/detail/detail.aspx?dbcode=CMFD&QueryID=7&CurRec=2&dbname=CMFD2012&filename=1012362862.nh&urlid=&yx=&v=MDU3MDlObktyWkViUElSOGVYMUx1eFlTN0RoMVQzcVRyV00xRnJDVVJMMmZadVp0RkNyaFZyM05WRjI2SExDK0g=

Yu JS, Shen KH, Chen WC, Her JS, Hsieh CL. Effects of electroacupuncture on benign prostate hyperplasia patients with lower urinary tract symptoms: a single-blinded, randomized controlled trial. Evidence-Based Complementary and Alternative Medicine. 2011, Article ID 303198, 8 pages doi:10.1155/2011/303198 .

Wang Y, Liu BY, Yu JN, Wu JN, Liu ZS. Electroacupuncture for moderate and severe benign prostatic hyperplasia: a randomized controlled trial. PLoS ONE. 2013;8, e59449. doi:10.1371/journal.pone.0059449.

Zheng RW. Electroacupuncture for benign prostatic hyperplasia-a randomised controlled trial and a MRS mechanism study. M.Sc. Thesis, Beijing University of Chinese medicine. 2016. Available from: http:// www.cnki.net/KCMS/detail/detail.aspx?dbcode=CDFD&QueryID=15&CurRec=1&dbname=CDFDLAST2016&filename=1016069128.nh&urlid=&yx=&v=MjYzNzkxRnJDVVJMMmZadVp0RkNyaFZiN0xWRjI2R0xPK0Y5RE9wNUViUElSOGVYMUx1eFlTN0RoMVQzcVRyV00=

Yang T. Effects of electroacupuncture for symptomatic benign prostatic hyperplasia. M.Sc. Thesis, China Academy of Chinese Medical Sciences. 2010. Available from: http:// www.cnki.net/KCMS/detail/detail.aspx?dbcode=CMFD&QueryID=19&CurRec=3&dbname=CMFD2011&filename=2010180441.nh&urlid=&yx=&v=MDkzNTdidkxWMTI2SHJLd0h0WElycEViUElSOGVYMUx1eFlTN0RoMVQzcVRyV00xRnJDVVJMMmZadVp0RkNyaFY=

Yu XH. The clinical curative effect obervation on treating benign prostatic hyperplasia with electro-acupuncture. M.Sc. Thesis, Hei Long Jiang Traditional Chinese Medicine University. 2005. Available from: http:// www.cnki.net/KCMS/detail/detail.aspx?dbcode=CMFD&QueryID=19&CurRec=9&dbname=CMFD0506&filename=2005120538.nh&urlid=&yx=&v=MTgyNjg3RGgxVDNxVHJXTTFGckNVUkwyZlp1WnRGQ3JoVzc3TVYxMjdHN0s2SHRUUHA1RWJQSVI4ZVgxTHV4WVM=

Wang K. Observation on therapeutic effect of electroacupuncture for benign prostatic hyperplasia. M.Sc. Thesis, Hei Long Jiang Traditional Chinese Medicine University .2006. Available from: http:// www.cnki.net/KCMS/detail/detail.aspx?dbcode=CMFD&QueryID=19&CurRec=8&dbname=CMFD0506&filename=2006113971.nh&urlid=&yx=&v=MTAxOTgzS1YxMjdHTEs1SGRqTHJwRWJQSVI4ZVgxTHV4WVM3RGgxVDNxVHJXTTFGckNVUkwyZlp1WnRGQ3JoVzc=

Du L. Abdomen acupuncture for benign prostatic hyperplasia. Journal of integrated traditional Chinese medicine and western medicine. 2012; 21:1637-1638.
