# Supplementary material for: Acupuncture for benign prostatic hyperplasia: A systematic review and meta-analysis
Source: PLoS One. 2017 Apr 4;12(4):e0174586. doi: 10.1371/journal.pone.0174586 (PMC5380320; doi:10.1371/journal.pone.0174586)
Supplement: S2 File — (DOC) [file pone.0174586.s004.doc]

List of full-text excluded articles (titles and reasons)

Reasons for exclusion: 1 not RCT; 2 other reasons (invalidated interventions for experimental or control group; follow-up shorter than 2 weeks)

| **Number** | **Title** | **Resources** | **Reasons for exclusion** |
| --- | --- | --- | --- |
| 1 | 90 cases of benign prostatic hyperplasia treated by laser acupuncture. | Academic journal of Bai Qiuen University, 1991. | 1 |
| 2 | 60 cases of benign prostatic hyperplasia treated by magnetic acupuncture. | Shanxi TCM Journal, 1993. | 1 |
| 3 | Therapeutic effect of urinary retention due to benign prostatic hyperplasia by combining electric acupuncture to Ciliao and  Zhongliao Acupoints with ultra short Wave Therapy. | Journal of Zhejiang College of TCM Vol.33 No.4 June.2009. | 1 |
| 4 | Clinical observation on electro-acupuncture for urinary retention of benign prostatic hyperplasia. | Shanghai TCM Journal, 2008. | 1 |
| 5 | Electro-acupuncture for symptomatic benign prostatic hyperplasia. | Journal of Chinese clinical physician, 2002. | 1 |
| 6 | Clinical observation on auricular acupuncture for benign prostatic hyperplasia. | Chinese journal of acupuncture and moxibustion, 2000. | 1 |
| 7 | Fire needle for benign prostatic hyperplasia. | Acupuncture academic journal, 1992. | 1 |
| 8 | 14 cases of benign prostatic hyperplasia treated by elongated needle and seeds embedding. | Journal of clinical acupuncture and moxibustion, 1994. | 1 |
| 9 | Clinical observation of benign prostatic hyperplasia treated by elongated needle. | Journal of clinical acupuncture and moxibustion, 2005. | 1 |
| 10 | 86 cases of benign prostatic hyperplasia treated by elongated needle. | Journal of clinical acupuncture and moxibustion, 1994. | 1 |
| 11 | Clinical observation of acupuncture for urinary retention due to benign prostatic hyperplasia. | Journal of clinical acupuncture and moxibustion, 1995. | 1 |
| 12 | 30 cases of benign prostatic hyperplasia treated by acupuncture and Chinese herbs. | Journal of clinical acupuncture and moxibustion, 1998. | 1 |
| 13 | Acupuncture and Chinese medicine for benign prostatic hyperplasia. | Acupuncture academic journal, 1994. | 1 |
| 14 | 30 cases of benign prostatic hyperplasia treated by acupuncture. | Shanxi TCM journal, 1994. | 1 |
| 15 | Effect of electro-acupuncture on nocturnal enuresis in patients with benign prostatic hyperplasia. | Shanghai acupuncture and moxibustion journal, 2014. | 1 |
| 16 | 41 cases of benign prostatic hyperplasia treated by electro-acupuncture. | Journal of clinical acupuncture and moxibustion, 2005. | 1 |
| 17 | 41 cases of benign prostatic hyperplasia treated by elongated needle. | Shanghai acupuncture and moxibustion journal, 2014. | 1 |
| 18 | 106 cases of benign prostatic hyperplasia and prostatitis treated by elongated needle. | Journal of clinical acupuncture and moxibustion, 1994. | 1 |
| 19 | 38 cases of benign prostatic hyperplasia treated by elongated needle. | Journal of Chengde medical college, 2002. | 1 |
| 20 | 37 cases of benign prostatic hyperplasia in seniors treated by acupuncture. | Shanghai acupuncture and moxibustion journal, 2005. | 1 |
| 21 | Clinical observation of eIectro-acupuncture at Ciliao and Huiyang acupoints and faradizations for urinary retention due to prostatic hyperplasia. | Shanghai acupuncture and moxibustion journal, 2008. | 1 |
| 22 | Acupuncture for nocturnal enuresis in benign prostatic hyperplasia patients. | Shanghai acupuncture and moxibustion journal, 2009. | 1 |
| 23 | 65 cases of benign prostatic hyperplasia treated by electro-acupuncture. | Journal of clinical acupuncture and moxibustion, 2005. | 1 |
| 24 | 65 cases of benign prostatic hyperplasia treated by electro-acupuncture and moxibustion. | Shanghai acupuncture and moxibustion journal, 2015. | 1 |
| 25 | 35 cases of benign prostatic hyperplasia treated by fire needle. | Shanghai acupuncture and moxibustion journal, 2014. | 1 |
| 26 | Clinical research of acupuncture for benign prostatic hyperplasia. | Jiangxi TCM journal, 2009. | 1 |
| 27 | Case report of acupuncture for benign prostatic hyperplasia. | Asia．Pacific Traditional Medicine, 2016 | 1 |
| 28 | 24 cases of benign prostatic hyperplasia treated by acupuncture and Mongolia medicine. | China ethnic medical journal, 2005. | 1 |
| 29 | 50 cases of benign prostatic hyperplasia treated by acupuncture and Mongolia medicine. | China ethnic medical journal, 2013. | 1 |
| 30 | Joint acupuncture treatment used for Conprosta Uganda patients with benign prostate hyperplasia of the clinical observation. | China foreign medical treatment, 2012. | 1 |
| 31 | “TongJinguaYu，KaiGanLiJi” acupuncture therapy in the treatment of benign prostatic hyperplasia of 50 cases. | Journal of clinical acupuncture and moxibustion, 2013. | 1 |
| 32 | Clinical observation on acupuncture with moxibustion for benign prostatic hyperplasia. | Journal of Tianjin University if TCM, 2002. | 1 |
| 33 | 30 cases of benign prostatic hyperplasia kidney deficiency syndrome treated by acupuncture and moxibustion. | Chinese journal of modern drug application, 2014. | 1 |
| 34 | Case report of acupuncture for benign prostatic hyperplasia. | World Chinese medicine, 2011. | 1 |
| 35 | 36 cases of benign prostatic hyperplasia treated by acupuncture and microwave therapy. | Journal of emergent TCM, 2008. | 1 |
| 36 | 50 cases of benign prostatic hyperplasia kidney Yang deficiency syndrome treated by acupuncture and moxibustion. | Chinese journal of acupuncture and moxibustion, 2004. | 1 |
| 37 | 23 cases of benign prostatic hyperplasia treated by acupuncture and microwave therapy. | Chinese and foreign medical research, 2011. | 1 |
| 38 | 37 cases of benign prostatic hyperplasia treated by acupuncture and moxibustion. | Journal of clinical acupuncture and moxibustion, 2004. | 1 |
| 39 | 41 cases of benign prostatic hyperplasia treated by acupuncture. | Chinese journal of ethnomedicine and ethnopharmacy, 2011. | 1 |
| 40 | 37 cases of benign prostatic hyperplasia in seniors treated by acupuncture. | Shanghai acupuncture and moxibustion journal, 2005. | 1 |
| 41 | 12 cases of benign prostatic hyperplasia in seniors treated by acupuncture. | Chinese journal of acupuncture and moxibustion, 2003. | 1 |
| 42 | Clinical study on acupuncture for benign prostatic hyperplasia. | Chinese journal of acupuncture and moxibustion, 2003. | 1 |
| 43 | 37 cases of benign prostatic hyperplasia treated by acupuncture. | China external therapy journal, 2014. | 1 |
| 44 | 60 cases of benign prostatic hyperplasia treated by acupuncture. | Journal of practical traditional Chinese internal medicine, 2004. | 1 |
| 45 | 20 cases of benign prostatic hyperplasia treated by acupuncture. | China applicable traditional and modern medicine journal, 2015. | 1 |
| 46 | Case report of benign prostatic hyperplasia with urinary retention treated by acupuncture. | Medical journal of people’s liberation army, 2007. | 1 |
| 47 | 20 cases of benign prostatic hyperplasia in middle-aged and seniors treated by acupuncture. | Chinese community physician journal, 2012. | 1 |
| 48 | Case report of benign prostatic hyperplasia treated by acupuncture. | Shanghai acupuncture and moxibustion journal, 2005. | 1 |
| 49 | Clinical observation on the treatment of benign prostatic hyperplasia by acupuncture combined with Chinese medicine. | Chinese journal of acupuncture and moxibustion（Electronic Edition），2014. | 1 |
| 50 | Observation on therapeutic effect of 34 cases of senile hyperplasia of prostate treated with acupuncture and moxibustion. | Chinese journal of acupuncture and moxibustion, 2001. | 1 |
| 51 | 67 cases of benign prostatic hyperplasia treated by acupuncture. | Internal Mongolia TCM journal, 2005. | 1 |
| 52 | 32 cases of benign prostatic hyperplasia treated by acupuncture. | Shanghai acupuncture and moxibustion journal, 2002. | 1 |
| 53 | 36 cases of benign prostatic hyperplasia treated by acupuncture. | Guang Ming TCM journal, 2009. | 1 |
| 54 | A prospective, randomized pilot trial of acupuncture of the kidney-bladder distinct meridian for lower urinary tract symptoms. | The Journal of urology, 2003. | 1 |
| 55 | Efficacy of electro-acupuncture in treating patients with benign prostatic hyperplasia. | Chinese journal of integrated traditional and western medicine, 2008. | 1 |
| 56 | Electro-acupuncture at BL 32 and BL 33 with microwave therapy for urinary retention due to benign prostatic hyperplasia. | Journal of clinical acupuncture and moxibustion, 2008. | 1 |
| 57 | Treatment of 123 cases of benign prostatic hyperplasia patients with magnetic plum-blossom needles. | Shanghai acupuncture and moxibustion journal, 2005. | 1 |
| 58 | Acupuncture at special points for urinary retention due to benign prostatic hyperplasia. | China rural medicine journal, 2015. | 1 |
| 59 | Scalp electro-acupuncture with Chinese herbs for benign prostatic hyperplasia. | Shanghai acupuncture and moxibustion journal, 2011. | 1 |
| 60 | Acupuncture with warm needles for benign prostatic hyperplasia. | Hu Bei journal of TCM, 2011. | 1 |
| 61 | Efficacy of eectroacupuncture in treating 103 patients with benign prostatic hyperplasia | Chinese journal of traditional and western medicine, 2008. | 1 |
| 62 | Therapeutic evaluation of acupuncture for benign prostatic hyperplasia patients. | Chinese journal of rehabilitation medicine, 2008. | 1 |
| 63 | Abdomen acupuncture with Chinese herbs for patients with benign prostatic hyperplasia. | Chinese clinical physicians, 2012. | 1 |
| 64 | 41 cases of benign prostatic hyperplasia treated by auricular acupuncture and elongated needle. | Journal of clinical acupuncture and moxibustion, 1997. | 1 |
| 65 | Clinical observation of fire needle for benign prostatic hyperplasia. | Journal of clinical acupuncture and moxibustion, 2012. | 1 |
| 66 | Clinical observation of electro-acupuncture with heat-sensitive moxibustion for benign prostatic hyperplasia. | Journal of clinical acupuncture and moxibustion, 2016. | 1 |
| 67 | 66 cases of benign prostatic hyperplasia treated by magnetic acupuncture. | Shan Xi TCM journal, 1997. | 1 |
| 68 | Clinical study of acupuncture at BL 33 and BL 34 with microwave therapy for benign prostatic hyperplasia. | Academic journal of Zhejiang TCM university, 2009. | 2 |
| 69 | Electro-acupuncture for benign prostatic hyperplasia-a randomized controlled trial. | Acupuncture and massage journal, 2011. | 2 |
| 70 | Clinical research of body and scalp acupuncture for benign prostatic hyperplasia. | World acupuncture journal, 2009. | 2 |
| 71 | The acupoints’ specificity research of acupuncture at BL 33 for benign prostatic hyperplasia. | Chinese journal of acupuncture and moxibustion, 2011. | 2 |
| 72 | Electro-acupuncture at CV 4 for benign prostatic hyperplasia-a randomized controlled trial. | Chinese journal of acupuncture and moxibustion, 2009. | 2 |
| 73 | Clinical observation on electro-acupuncture for benign prostatic hyperplasia. | M.Sc. Thesis, Hei Long Jiang Traditional Chinese Medicine University. 2010. | 2 |
| 74 | Therapeutic observation on acupuncture for benign prostatic hyperplasia. | Chinese journal of rehabilitation medicine, 2008. | 2 |
| 75 | Clinical study of electro-acupuncture for benign prostatic hyperplasia. | Journal of Liao Ning university of TCM, 2006. | 2 |
| 76 | Acupuncture and moxibustion in the treatment of benign prostatic hyperplasia (kidney deficiency and blood stasis) clinical observation. | M.Sc. Thesis, Hei Long Jiang Traditional Chinese Medicine University. 2015. | 2 |
| 77 | Clinical research of warm needle and acupoint thread embedding for benign prostatic hyperplasia patients. | Shanxi TCM journal, 2014. | 2 |
| 78 | Clinical research of scalp needle and elongated needle for benign prostatic hyperplasia patients. | Journal of Tian Jin university of TCM, 2002. | 2 |
| 79 | The clinical study on treatment of benign prostatic hyperplasia with acupuncture and microwave. | M.Sc. Thesis, Shan Dong Traditional Chinese Medicine University. 2008. | 2 |
| 80 | Clinical observation on the effect of thermal moxibustion combined with electro-acupuncture in the treatment of benign prostatic hyperplasia. | Journal of clinical acupuncture and moxibustion, 2016. | 2 |
| 81 | Clinical observation of benign prostatic hyperplasia treated with scalp acupuncture and body acupuncture. | World journal of acupuncture and moxibustion, 2009. | 2 |
| 82 | Clinical observation of scalp needle and elongated needle for benign prostatic hyperplasia patients. | Journal of Liao Ning university of TCM, 2007. | 2 |
| 83 | Clinical research on acupuncture and navel filling therapy for kidney function of benign prostatic hyperplasia patients. | China and foreign treatment, 2014. | 2 |
| 84 | Clinical research of embedded needles therapy in treating benign prostatic hyperplasia. | M.Sc. Thesis, Guang Zhou Traditional Chinese Medicine University. 2008. | 2 |
| 85 | Observation of 182 cases of senile benign prostatic hyperplasia by mini fire needles. | Chinese TCM information journal, 2011. | 2 |
| 86 | Treatment of 149 cases of prostate hyperplasia by magnetic plum-blossom needle composite treatment. | Shanghai acupuncture and moxibustion journal, 2005. | 2 |
| 87 | Clinical on hyperplasia of prostate (moist heat and blood stasis) with acupuncture added Chinese herbs. | M.Sc. Thesis, Guang Zhou Traditional Chinese Medicine University. 2010. | 2 |
| 88 | Clinical efficacy of acupuncture combined with oral Yishen Huoxue Decoction in treatment of elderly prostatic hypertrophy patients. | Chinese archives of traditional Chinese medicine, 2014. | 2 |
| 89 | Clinical research on warm needling moxibustion combined with Chinese herbs for chronic prostatic hyperplasia. | M.Sc. Thesis, Guang Zhou Traditional Chinese Medicine University. 2010 | 2 |
| 90 | Abdomen acupuncture and Chinese medicine for 46 cases of benign prostatic hyperplasia. | Chinese clinical practitioners, 2012. | 2 |
| 91 | Electroacupuncture at Zhongji (CV 3) for treatment of benign hyperplasia of prostate: a multi central randomized controlled study | Chinese journal of acupuncture and moxibustion, 2009. | 2 |
| 92 | To observe the clinical therapeutic effect of electro-acupuncture on benign prostatic hyperplasia. | M.Sc. Thesis, Hei Long Jiang Traditional Chinese Medicine University. 2010. | 2 |
| 93 | The clinical curative observation in treating benign prostatic hyperplasia with electro-acupuncture. | M.Sc. Thesis, Hei Long Jiang Traditional Chinese Medicine University. 2010. | 2 |
| 94 | Observation on therapeutic effects of elongated needle therapy on dysuria induced by benign prostatic hyperplasia. | Chinese journal of acupuncture and moxibustion, 2008. | 2 |
| 95 | Han’s acupuncture for urinary discharging difficulty due to benign prostatic hyperplasia-a multi-centered randomized controlled trial. | China TCM technology, 2005. | 2 |
| 96 | Efficacy of electroacupuncture at Zhongliao point (BL33) for mild and moderate benign prostatic hyperplasia: study protocol for a randomized controlled trial. | Trials, 2011 | 2 |
| 97 | Clinicalvobservation on treatment of prostatic hyperplasia by placement of polepiece on acupoints. | Chinese journal of acupuncture and moxibustion, 2003. | 2 |
| 98 | Analysis of treating 60 cases of hyperplasia of prostate by acupuncture. | Journal of practical traditional Chinese internal medicine, 2004. | 2 |
| 99 | Therapeutic effect of acupuncture combined with microwave radiation at acupoints on benign prostatic hyperplasia. | Chinese journal of acupuncture and moxibustion, 2004. | 2 |
| 100 | The effect observation on hyperplasia of prostate treated mainly by warming-needle. | Journal of Tianjin University of TCM, 2002. | 2 |
| 101 | Clinical observation on treating hyperplasia of prostatemainly with awn needle. | Journal of clinical acupuncture and moxibustion, 2005. | 2 |
| 102 | Observations on the efficacy of electro-magnetotherapy plus warming acupuncture for treating 61 patients with benign prostate hyperplasia. | Shanghai acupuncture and moxibustion journal, 2006. | 2 |
| 103 | The clinical observation of acupuncture and Chinese medicinal enema for prostatic hyperplasia. | Shanghai acupuncture and moxibustion journal, 2015. | 2 |
| 104 | Clinical observations on scalp electro-acupuncture plus medication for the treatment of benign prostatic hyperplasia. | Shanghai acupuncture and moxibustion journal, 2011. | 2 |
| 105 | Clinical observation on electro-acupuncture for benign prostatic hyperplasia. | Seek medical and ask the medicine, 2011. | 2 |
| 106 | Electro-acupuncture for benign prostatic hyperplasia. | Chinese senile medicine journal, 2011. | 2 |
| 107 | Fast acupuncture and moxibustion for benign prostatic hyperplasia. | Chinese journal of acupuncture and moxibustion, 2008. | 2 |
| 108 | Auricular acupuncture and ultrasonic infrared therapy for benign prostatic hyperplasia. | Chinese journal of acupuncture and moxibustion, 2000. | 2 |
| 109 | Observation on the comparison in therapeutic effects between the 650 nm laser acupoint irradiation and acupuncture on the treatment of benign prostatic hyperplasia. | ACTA laser biology SINICA, 2007. | 2 |
| 110 | A clinical study of 108 cases of benign prostatic hyperplasia by acupuncture and Chinese medicine. | Academic journal of Chinese medicine, 2006. | 2 |
| 111 | Clinical research of acupuncture plus Chinese medicinal enema for benign prostatic hyperplasia. | Journal of practical traditional Chinese medicine, 2011. | 2 |
| 112 | A clinical research of acupuncture and Chinese medicine for benign prostatic hyperplasia. | Shanghai acupuncture and moxibustion journal, 2007. | 2 |
| 113 | Therapeutic observation of acupuncture plus Chinese medicinal enema for benign prostatic hyperplasia. | Shanghai acupuncture and moxibustion journal, 2014 | 2 |
| 114 | Acupuncture and Chinese medicine application for benign prostatic hyperplasia. | Journal of clinical acupuncture and moxibustion, 2004. | 2 |
| 115 | Acupuncture and aupoint-injection for urinary retention after surgery for benign prostatic hyperplasia. | Hebei TCM journal, 2010. | 2 |
| 116 | Clinical study of acupuncture and western medicine for benign prostatic hyperplasia. | Henan medical research, 2013. | 2 |
| 117 | Therapeutic effect of acupuncture combined with microwave radiation at acupoints on benign prostatic hyperplasia | Chinese journal of acupuncture and moxibustion, 2004. | 2 |
| 118 | Clinical study of acupuncture and moxibustion for benign prostatic hyperplasia. | Hu Bei journal of TCM, 2013. | 2 |
| 119 | Clinical efficacy of needle warming moxibustion and proscar in the treatment of elder benign prostatic hyperplasia. | Chinese journal of prevention and nutrition, 2012. | 2 |
| 120 | Clinical study of acupuncture with thread embedding therapy for treatment of benign prostatic hyperplasia. | Shanxi TCM journal, 2014. |  |
| 121 | Tamsulosin combined with acupuncture for treatment of benign prostatic hyperplasia. | Academic journal of Guangzhou medical university. | 2 |
| 122 | Clinical study of acupuncture and surgery for benign prostatic hyperplasia. | Modern journal of integrated traditional Chinese medicine and western medicine, 2006. | 2 |
| 123 | Acupuncture and massage for 61 cases of benign prostatic hyperplasia. | Journal of applicable medicine, 2008. | 2 |
| 124 | Electro-acupuncture and acupoint injection for benign prostatic hyperplasia. | Medical information, 2014. | 2 |
| 125 | Acupuncture and Jin Gui Shen Qi Pills for benign prostatic hyperplasia. | National journal of andrology, 2007. | 2 |
| 126 | Acupuncture and navel filling therapy for benign prostatic hyperplasia. | Medical information, 2013. | 2 |
| 127 | Clinical observation of benign prostatic hyperplasia treated with electro-acupuncture combined with Jingzhu Qianlie Longbitong capsules. | Journal of clinical acupuncture and moxibustion, 2014. | 2 |
| 127 | Clinical study on acupuncture and electro-magnetic treatment for benign prostatic hyperplasia. | Shanghai acupuncture and moxibustion journal, 2006. | 2 |
| 129 | Electro-acupuncture with bee venom suppositories for benign prostatic hyperplasia. | Journal of clinical acupuncture and moxibustion, 2003. | 2 |
| 130 | Clinical study on treating of benign prostatic hyperplasia with urinary retention by acupuncture and massage. | Journal of Jiangxi university of TCM, 2008. | 2 |
| 131 | Clinical study on treating of benign prostatic hyperplasia (blood stagnating syndrome) with acupuncture and medicine. | Clinical journal of Chinese and modern medicine, 2012. | 2 |
| 132 | Clinical study on treating of benign prostatic hyperplasia with puncturing points on foot Jueyin. | Modern hospital 2005. | 2 |
| 133 | Clinical observation of elongated needle for benign prostatic hyperplasia. | Journal of clinical acupuncture and moxibustion, 2005. | 2 |
| 134 | Clinical observation of electro-acupuncture for benign prostatic hyperplasia. | Shanghai acupuncture and moxibustion journal, 2007. | 2 |
| 135 | Clinical effect observation of turtle probing needling in the treatment of benign prostatic hyperpiesia. | China modern medicine,2016 | 2 |
| 136 | Clinical observation on acupuncture for benign prostatic hyperplasia. | Chinese journal of acupuncture and moxibustion, 2014. | 2 |
| 137 | Clinical observation of acupuncture plus moxibustion for benign prostatic hyperplasia. | Shanghai acupuncture and moxibustion journal, 2015. | 2 |
| 138 | Electro-acupuncture at ZhoneJi(CV 3) for treatment of benign hyperplasia of prostate：A multi-centered randomized controlled study. | Chinese journal of acupuncture and moxibustion, 2008. | 2 |
| 139 | The efficacy of electro-acupuncture and traditional Chinese medicine in the clinical treatment of benign prostate hyperplasia. | China modern doctor, 2011. | 2 |
