# Supplementary material for: Acupuncture for benign prostatic hyperplasia: A systematic review and meta-analysis
Source: PLoS One. 2017 Apr 4;12(4):e0174586. doi: 10.1371/journal.pone.0174586 (PMC5380320; doi:10.1371/journal.pone.0174586)
Supplement: S1 Table — (DOC) [file pone.0174586.s005.doc]

Table –Description of studies included in the review

| Study | N randomised | Follow-up | Participants | Intervention | Review outcome measures |
| --- | --- | --- | --- | --- | --- |
| Liu 2012[18] | Acupuncture n=24  Sham acupuncture n=24 | Baseline, 4w, 3mo | Patients with diagnosed BPH for over 3 months ( IPSS≥8). | Acupuncture group:  Type: 20Hz electroacupuncture  Location: BL33 (bilateral).  Needle: Long needle 4-4.5cun(100mm or 125mm).  Control group:  Location: two points which are 2 cun (6.8 cm) lateral to BL33s. | International Prostate Symptom Score (IPSS),  Quality of life(QOL),  postvoid residual urine (PVR),  maximum flow rate (Qmax),  Adverse events: reported |
| Yu 2011 [19] | Acupuncture n=21  Sham acupuncture n=21 | Baseline, 6w. | BPH patients with IPSS > 8, and Qmax < 15mL/sec. | Acupuncture group:  Type: 2Hz electroacupuncture  Location:  (1) GV3-GV4 pair  (2) ST36-SP6 pair left  (3) ST36-SP6 pair right  Needle:2 cun (50mm)  Control group:  acupuncture needles were inserted into the subcutaneous tissue to a depth of 2 mm, the location was 1 cm lateral to the above-mentioned acupoints, | Qmax,  average flow rate (Qave),  total flow time,  total void volume,  IPSS,  prostate specific antigen(PSA)  Adverse events: not reported |
| Wang 2013 [20] | Acupuncture n=50  Sham acupuncture n=50 | Baseline, 6w,18w | Men with BPH and IPSS≥8. | Acupuncture group:  Type: 20Hz electroacupuncture  Location: BL33 (bilateral).  Needle: Long needle (100mm).  Control group：  Location: two points which are 2 cun lateral to BL33s. | IPSS,  PVR,  Qmax  Adverse events: reported |
| Zheng 2016 [21] | Acupuncture n=30  Tamsulosin hydrochloride sustained release capsules n=30 | Baseline, 6w | Men with confirmed diagnosis of BPH from urologists. | Acupuncture group:  Type: 5Hz electroacupuncture  Location: RN3-RN4 pair  Needle: 1.5 cun (40mm)  Control group: Tamsulosin hydrochloride sustained release capsules, oral, 0.2mg daily. | IPSS,  QOL,  Qmax,  PVR,  prostate volume (PV)  PSA  Adverse events: reported |
| Yang 2010 [22] | Acupuncture n=87  Terazosin hydrochloride tablets n=88 | Baseline, 4w, 6mo 18mo | BPH patients with 8≤I PSS≤20. | Acupuncture group:  Type: 20Hz electroacupuncture.  Location: BL33 (bilateral)  Needle: Long needle(100mm)  Control group:  Terazosin hydrochloride tablets, oral, 2mg daily. | IPSS,  Qmax,  PV,  PVR,  Bother of Score(BS),  Adverse events: reported |
| Yu 2005 [23] | Acupuncture n=48  Tamsulosin hydrochloride sustained release capsules n=44 | Baseline, 4w | Men with diagnosed BPH. | Acupuncture group:  Type: 20 Hz electroacupuncture.  Location: BL 35 (bilateral)  Needle:.2.5cun(65mm)  Control group:  Tamsulosin hydrochloride sustained release capsules, oral, 0.2mg daily. | IPSS,  BS,  PVR,  PV,  Qmax’  Adverse events: reported |
| Wang 2006 [24] | Acupuncture n=43  Finasteride n=41 | Baseline, 4w | Men with diagnosed BPH. | Acupuncture group:  Type: 20Hz electroacupuncture  Location: BL 35 (bilateral)  Needle: 2.5cun(65mm)  .  Control group:  Finasteride 5mg oral daily. | IPSS,  QOL,  PV,  PVR,  Qmax  Adverse events: reported |
| Du 2012 [25] | Acupuncture n=30  Finasteride n=30 | Baseline, 3mo | Men with BPH and IPSS≥8. | Acupuncture group:  Type: Hand acupuncture.  Location: RN4, RN6, RN9, RN12, ST25 (bilteral)  Needle: 1.5 cun (40 mm)  Control Group:  Finasteride 5mg oral daily. | IPSS,  QOL,  Qmax,  PVR,  PV  Adverse events: not reported |
